# External validation of radiobiological models for local control prediction in lung cancer patients treated with stereotactic body radiation therapy

**DOI:** 10.3389/fonc.2024.1431140

**Published:** 2025-01-10

**Authors:** Bao-Tian Huang, Pei-Xian Lin, Ying Wang, Li-Mei Luo

**Affiliations:** ^1^ Department of Radiation Oncology, Cancer Hospital of Shantou University Medical College, Shantou, China; ^2^ Department of Nosocomial Infection Management, The Second Affiliated Hospital of Shantou University Medical College, Shantou, China; ^3^ Department of Radiation Oncology, Affiliated Cancer Hospital & Institute of Guangzhou Medical University, Guangzhou, China

**Keywords:** external validation, radiobiological model, local control prediction, lung cancer, stereotactic body radiation therapy

## Abstract

**Background:**

The debate regarding the accuracy of radiobiological models for local control (LC) prediction in lung cancer patients undergoing stereotactic body radiation therapy (SBRT) remains unresolved. The study seeks to externally validate the predictive efficacy of radiobiological models using single-institutional SBRT database.

**Methods:**

The cohort comprised 153 patients diagnosed with primary or metastatic lung cancer who underwent SBRT. The study employed three radiobiological models to estimate the probability of 2-year LC, including the Liu model, Klement model, and Ohri model. Furthermore, the likelihood of 3-year LC was predicted using the Liu model, Klement model, Gucken model, and Santiago model. The performance of the prediction models was assessed through the AUC values of the receiver operating characteristic (ROC) curve and the calibration plots.

**Results:**

Local recurrence was observed in 38.6% of patients (59/153) within two years, and in 43.1% (66/153) within three years after the radiotherapy. The ROC curves indicated discriminative power for all the 2-year and 3-year models, with the exception of the Klement model. The Ohri model showed a significantly improved discriminative ability than the Klement model for 2-year prediction, while it was not statistically significant when compared to the Liu model. However, no significant differences were found among the four models in terms of 3-year LC prediction. The calibration plots, using the Hosmer-Lemeshow goodness-of-fit test, confirmed that the predicted probabilities of the models were in agreement with the actual observation with *P*>0.05, except for the 2-year LC prediction using the Klement model.

**Conclusion:**

Considering the balance between prediction accuracy and model simplicity, it is recommended to utilize the Ohri model for 2-year LC prediction and either the Gucken model or Santiago model for 3-year LC prediction.

## 1 Introduction

Lung cancer is the predominant cause of cancer-related deaths worldwide (1). Stereotactic body radiation therapy (SBRT) has gained widespread acceptance as a potent and tolerable therapeutic alternative for patients with early-stage non-small cell lung cancer (NSCLC) who are not candidates for surgical intervention (2). This treatment modality involves administering a higher radiation dose to the tumor target within a limited number of sessions, thereby achieving an elevated biologically effective dose (BED). The objective of SBRT is to optimize the therapeutic benefit while concurrently reducing the adverse effects on nearby organs at risk (OARs) (3, 4). Despite the encouraging outcomes associated with SBRT, local recurrence remains a challenge in certain cases. Research has indicated that the rate of recurrence following SBRT treatment is approximately 13.0% to 18.8% (5, 6). It is noteworthy that there are documented cases where patients who experience a local recurrence and subsequently undergo successful salvage treatment have survival outcomes comparable to those who received primary SBRT without any recurrence (7). Given these findings, it is of paramount importance to accurately identify patients who are more likely to respond positively to SBRT.

Currently, the application of a BED_10_ > 100 Gy stands as the most prevalent dose threshold for predicting local recurrence. This method is widely used for its effectiveness in translating into improved outcomes for tumor LC (8). To enhance the accuracy of prediction, various radiobiological models that incorporate both clinical and dosimetric factors have been introduced for predicting the 2-year and 3-year dose response after lung SBRT in recent years (9–14). Given the diverse range of doses and fractionation schemes applied in SBRT, the radiobiological models capable of translating the dosimetric change into radiobiological benefits are extensively employed to evaluate and compare the dose response across various fractionation regimens. These models work by employing mathematical analysis to predict the outcome for the tumor, using parameters that are determined from fitting clinical data. Despite the introduction of numerous radiobiological models, their accuracy has not been confirmed with external data sets, casting doubt on their clinical utility. It has been noted that there can be discrepancies of up to 15% when different prediction models are applied (15).

The objective of the current study is to compare the effectiveness of radiobiological models in predicting LC for lung cancer patients who have undergone SBRT, using the SBRT database available at our institution.

## 2 Materials and methods

### 2.1 Study population

The study cohort was comprised of 153 patients with either primary or secondary lung cancer. Each patient received SBRT at the Cancer Hospital of Shantou University Medical College between July 2011 and January 2021. The study was conducted in accordance with the principles of the Declaration of Helsinki and was granted approval by the relevant ethical board, where written informed consent was waived. The criteria for inclusion in the study were as follows: (1) A confirmed diagnosis of primary or secondary lung cancer that was treated using SBRT. (2) Complete baseline clinical information and subsequent follow-up data were available for all patients.

### 2.2 Radiotherapy treatment

Risk-adapted dose schedules were implemented for the treatment planning. Dose schedules frequently utilized, such as 12.5 Gy×4, 10.0 Gy×5, 25 Gy×1, 10.0 Gy×4, and 12.0 Gy×4, were chosen based on a compromise between tumor size and proximity to the OARs. For instance, the 12.5 Gy×4 dose schedule referred to a 50 Gy delivered in 4 fractions, with the other dose schedules being similarly defined. The Eclipse treatment planning system was utilized for the treatment planning (July 2011-September 2019,Version 10.0; October 2019-January 2021, Version 15.5, Varian Medical System, Inc., Palo Alto, CA). Each patient underwent treatment on a TrueBeam linear accelerator (LINAC), employing either the RapidArc or Intensity-Modulated Radiotherapy (IMRT) treatment technique. Fractional setup error was corrected with the aid of cone beam computed tomography (CBCT) equipped on the LINAC. Before performing the radiotherapy, fluoroscopic imaging on the LINAC was utilized to assess the movement of the tumor in certain patients, confirming that the trajectory of the tumor remained within the planned target volume (PTV).

### 2.3 Follow-up

Patients were subjected to CT scans on a bi-monthly basis during the initial year post-completion of their treatment. The interval for evaluation was then extended to semi-annual assessments (every six months) after the first year (16). The most recent follow-up was conducted in January 2024. LC of the tumor was characterized by the absence of any recurrence at the site where the treatment was administered. The primary method for establishing local recurrence predominantly involved a biopsy and an enhanced CT scan. This process identified a progressive increase in the size of the primary tumor or the area surrounding it, as observed on two successive CT scans with a minimum time gap of six months. A pathological examination was conducted when there was a strong suspicion of local recurrence. In cases where a biopsy sample was unattainable, the diagnosis was made using contrast-enhanced CT or PET/CT scans. Additionally, clinical symptoms and signs evaluated by oncologists could also be utilized as indicators to detect local recurrence (17).

### 2.4 Data collection

We gathered clinical attributes of the patients, encompassing factors such as gender, age, tumor location, clinical stage, whether the tumor was primary or metastatic, and tumor diameter. Radiotherapy-related parameters, including prescription dose recorded as BED (BEDPD), the maximum BED in the target (BEDD_max_), treatment fractions, and treatment duration were also collected. The linear-quadratic (LQ) model, utilizing an α/β ratio of 10 Gy, was employed to compute the BED. The formula for BED is expressed as, BED = *n* × *d* × [1 + *d*/(α/β)], where *n* denotes the fraction number and *d* represents the fractional dose.

### 2.5 Radiobiological models

We utilized the Liu model, Klement model, and Ohri model to calculate the 2-year probability of LC. For the estimation of the 3-year LC probability, we employed the Liu model, Klement model, Gucken model, and Santiago model. The Liu model accounts for the tumor regrowth locally following radiotherapy (12). It is applicable for predicting LC outcomes in relation to the duration of follow-up. The Klement model operates on a Bayesian framework for estimating the cure rate (10). It is also capable of determining the LC value as a function of the follow-up period. The Ohri model, Gucken model, and Santiago model are all logistic regression models that establish a logistic correlation between the tumor control probability (TCP) and the BED (13, 14, 18). For the computation of LC data, we employed a custom program developed on MATLAB R2021b (MathWorks, USA). All The parameters for the radiobiological models were sourced either from the original publications or directly from the authors by personal communication. The formula and model parameters for TCP calculation were detailed in the initial document and re-summarized in **Table 1**.

**Table 1:** The formula and model parameters of different radiobiological models.

Study	Models	TCP formula	Model parameters
Liu et al. (12)	Liu Model	BEDRegrowth=D(1+dα/β)−ln2τPΓα $t=\frac{e^{-[\alpha *BED-{\left(\frac{\ln2}{\tau_{P}}\tau \right)}^{\delta }]}-K_{cr}{/}_{K}^{0}}{\sigma_{k}/K_{0}}$ $TCP=1-\frac{1}{\sqrt{2\pi }}\int_{-\infty }^{t}e^{-\frac{x^{2}}{2}}dx$ , Where Γ is the elapsed treatment time, T_p_ is potential effective tumor doubling time, τ is the follow-up time after treatment, δ is parameter characterizing the speed of tumor cell regrowth after radiation treatment, T_p_ is the conventional doubling time, K_cr_ is the critical tumor number that defines the control of an individual tumor, σ_k_ is the Gaussian width for the distribution of tumor cell numbers.	**Table 1** of the original publication
Klement et al. (10)	Klement model	$TCP={\text{e}}^{\left(-\theta (1-S(t))\right)}$ $S(\text{t})={\text{e}}^{\left(-t^{\rho }e^{\eta }\right)}$ , Where θ is the cure parameter, t is the follow-up time after treatment. Before entering the model, binary covariates were rescaled to ±0.5 and continuous covariates were standardized to have mean 0 and standard deviation 0.5.	**Table 2** of the original publication, CIS99 model
Ohri et al. (18)	Ohri model	$TCP=e^{(BED_{10}-c*L-TCD50)/k}÷(1+e^{(BED_{10}-c*L-TCD50)/k})$	c=10 Gy/cm TCD50 = 0 Gy k=31 Gy
Gucken et al. (14)	Gucken model	$TCP=e^{(BED_{10}-TCD50)/k}÷(1+e^{(BED_{10}-TCD50)/k})$	TCD50=-1 Gy k=80 Gy
Santiago et al. (13)	Santiago model	$TCP={\text{e}}^{(BED_{10}-TCD50)/k}÷(1+{\text{e}}^{(BED_{10}-TCD50)/k})$	TCD50=-60.2 Gy k=113.3 Gy

TCP, Tumor control probability.

### 2.6 Dose-response visualization of different radiobiological models

To graphically represent the variance in dose-response among different radiobiological models, a 3D map was conceptualized, assuming a scenario with 3 treatment fractions, to illustrate the interconnection between TCP, BED, and tumor size. The development of this 3D map was executed using a program that was custom-designed on the MATLAB platform.

### 2.7 Model performance comparison and statistical analysis

The discriminative ability of the models was evaluated using the area under the curve (AUC) values derived from the receiver operating characteristic (ROC) curve, which were charted using MedCalc software (MedCalc, Version 20.015, MedCalc Software Ltd). *P<*0.05 for the ROC curve indicated a statistically significant ability to discriminate for the model. Furthermore, the DeLong test comparison with *P*<0.05 between the two models revealed a statistically significant difference. The calibration ability of the models refers to its capability to generate predictions that align well with the actual outcomes. Model calibration was carried out using the Hosmer-Lemeshow test and *P*>0.05 interpreted as indicating no discrepancy between the observed and the predicted probabilities. The calibration of the model was also depicted by plotting the observed versus the predicted probabilities. A calibration curve of 45-degrees indicates a perfect prognostic prediction (10). If the plot lies below the line, the model is under-calibrated (or too conservative), meaning it predicts a lower probability than the actual observed rate. Conversely, if the plot lies above the line, the model is over-calibrated (or too confident), predicting a higher probability than the actual observed rate. Additionally, the accuracy, sensitivity, and specificity of the models were compared. The sensitivity and specificity values were directly retrieved from the MedCalc software, whereas the accuracy values were computed using the recommended threshold values provided by the software. Moreover, a confusion matrix was used for presenting the prediction accuracy of the radiobiological models.

## 3 Results

### 3.1 3D visualization of the dose-response relationship across radiobiological models

**Figure 1:**
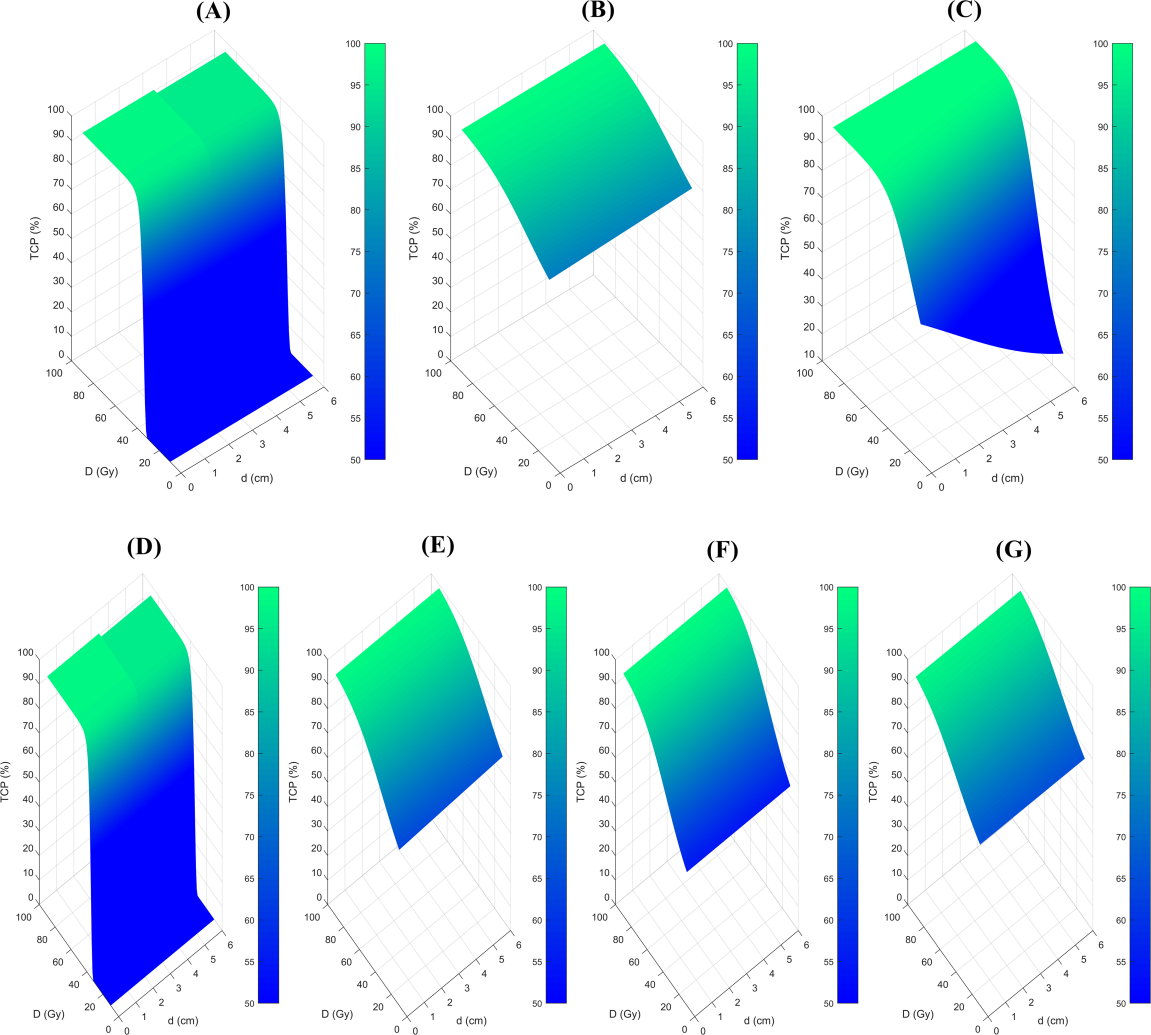
3D map of the relationship between the TCP, BED, and tumor size among different radiobiological models. **(A)**, Liu model for 2-year LC prediction, **(B)**, Klement model for 2-year LC prediction, **(C)**, Ohri model for 2-year LC prediction, **(D)**, Liu model for 3-year LC prediction, **(E)**, Klement model for 3-year LC prediction, **(F)** Gucken model for 3-year LC prediction, and **(G)**, Santiago model for 3-year LC prediction. TCP, Tumor control probability.

**Figure 1** showed the 3D map illustrating the relationship between TCP, BED, and tumor size across various radiobiological models. Notably distinct dose-response relationships were observed, regardless of whether the predictions were for 2-year (**Figures 1A–C**) or 3-year LC (**Figures 1D–G**). The z-axis represents the TCP value, while the x and y axes correspond to the treatment dose and tumor size, respectively. Typically, when employing the same TCP range, the shape of the figures should be similar among different models. However, in this case, despite applying a consistent TCP range of 50% to 100% across all models, the plots displayed substantial variation, suggesting that the predicted outcomes of the radiobiological models showed discrepancies. This becomes especially apparent for the 2-year prediction models.

### 3.2 Patient characteristics and treatment outcome

The study encompassed a total of 153 lung cancer patients who fulfilled the inclusion criteria, supplying complete clinical data along with extensive follow-up details. The characteristics of the patients’ treatment were presented in **Table 2**. By the time of the final follow-up, 38.6% of the patients (59/153) had encountered local recurrence within two year following the radiotherapy treatment. Additionally, 43.1% of the patients (66/153) had experienced local recurrence a three-year period after the radiotherapy treatment.

**Table 2:** Patient and treatment characteristics in this study.

Characteristic	Counts (%)/Median (IQR)
Gender	
Male	115 (75.2)
Female	38 (24.8)
Age	65.0 (16.0)
Clinical stage	
I	48 (31.4)
II	14 (9.2)
III	10 (6.5)
IV	81 (52.9)
Primary or metastatic	
Primary	93 (60.8)
Metastatic	60 (39.2)
Tumor location	
Peripheral	133 (86.9)
Central	20 (13.1)
Tumor diameter (cm)	3.2 (2.6)
BEDPD (Gy)	95.2 (25.6)
BEDD_max_ (Gy_10_)	114.4 (41.1)
Fractions	4.0 (3.0)
Treatment duration (days)	7.0 (7.0)
2-year LC	
Yes	94 (61.4)
No	59 (38.6)
3-year LC	
Yes	87 (56.9)
No	66 (43.1)
Dose schedules^*^	
12.5 Gy×4	27 (17.6%)
10.0 Gy×5	17 (11.1%)
25.0 Gy×1	15 (9.8%)
10.0 Gy×4	15 (9.8%)
12.0 Gy×4	8 (5.2%)
24.0 Gy×1	6 (3.9%)
Others	65 (42.6%)

BEDPD=The prescribed dose recorded as BED. BEDD_max_=The maximum dose in PTV recorded as BED.

IQR, Interquartile range.

^*^Dose schedules were recorded as counts (%).

### 3.3 The probability predicted by the 2-year and 3-year radiobiological models

**Table 3** provided a summary of the LC probability data for 153 patients as predicted by the 2-year and 3-year radiobiological models. The median probability data for both the 2-year and 3-year prediction models demonstrated significant differences across the various models. It is important to highlight that the Klement model projected an 8.4% reduction in the 3-year LC data when compared to the 2-year LC data. Conversely, the Liu model suggested a minor decline in the LC data as it transitioned from a 3-year to a 2-year timeframe.

**Table 3:** The LC probability predicted by the 2-year and 3-year models.

Endpoint	Models	Median (IQR) (%)
2-year LC	Liu Model	95.4 (6.8)
	Klement model	81.5 (14.0)
	Ohri model	93.6 (13.2)
3-year LC	Liu Model	95.4 (8.2)
	Klement model	73.1 (19.1)
	Gucken model	80.9 (8.2)
	Santiago model	82.4 (5.4)

LC, Local control; IQR, Interquartile range.

### 3.4 Model performance evaluation

**Figure 2:**
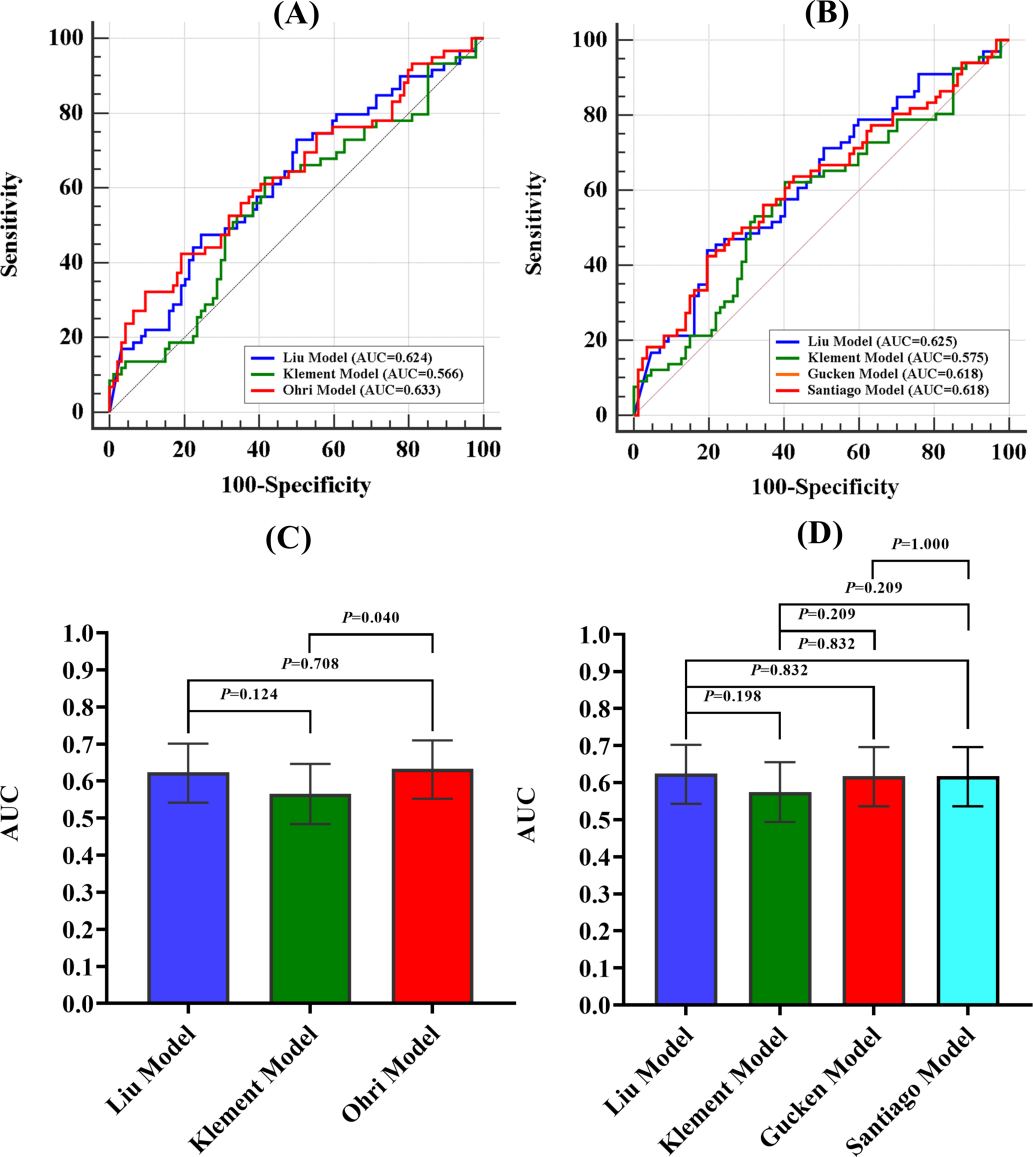
ROC curves for 2-year and 3-year LC prediction and AUC comparison for the 2-year and 3-year prediction models. **(A)** ROC curves for 2-year prediction model, **(B)** ROC curves for 3-year prediction model, **(C)** AUC comparison for the 2-year prediction models, **(D)** AUC comparison for the 3-year prediction model. AUC, Area Under the Curve.

The discriminative ability of the models was examined in terms of the AUC value. The AUC values of the models were presented in **Table 4**. For 2-year LC prediction, the AUC values for the Liu model, Klement model, and Ohri model were 0.624 (95% CI 0.542-0.701), 0.566 (95% CI 0.484-0.646), and 0.633 (95% CI 0.552-0.710), respectively. For the 3-year LC prediction, the AUC values for the Liu model, Klement model, Gucken model, and Santiago model were 0.625 (95% CI 0.543-0.702), 0.575 (95% CI 0.494-0.655), 0.618 (95% CI 0.536-0.696), and 0.618 (95% CI 0.536-0.696), respectively. All models, for both 2-year and 3-year predictions, exhibited discriminative ability (*P*<0.05 for the ROC curve), with the exception of the Klement model (*P*>0.05 for the ROC curve). The ROC curves for the 2-year and 3-year LC predictions were depicted in **Figures 2A, B**.

**Table 4:** Performance of the 2-year and 3-year LC prediction models.

Endpoint	Models	AUC (95% CI)	*p*-value	Accuracy (%)	Sensitivity (%)	Specificity (%)
2-year LC	Liu Model	0.624 (0.542-0.701)	0.0085	64.7	47.5	75.5
	Klement model	0.566 (0.484-0.646)	0.174	59.5	62.7	58.5
	Ohri model	0.633 (0.552-0.710)	0.0049	66.0	42.4	80.9
3-year LC	Liu Model	0.625 (0.543-0.702)	0.0064	64.7	43.9	80.5
	Klement model	0.575 (0.494-0.655)	0.113	60.1	62.1	59.8
	Gucken model	0.618 (0.536-0.696)	0.0112	63.4	42.4	80.5
	Santiago model	0.618 (0.536-0.696)	0.0112	63.4	42.4	80.5

LC, Local control.

The criterion values recommended by the MedCalc software for accuracy calculation for the 2-year prediction model were 0.9481, 0.8145, and 0.8673, respectively. And the corresponding value for 3-year prediction were 0.9379, 0.7310, 0.7655, and 0.7954, respectively.

The Ohri model showed improved discriminative ability in comparison to the Klement model for 2-year prediction (*P*<0.05). However, no statistically significant difference was observed when it was compared to the Liu model (*P*>0.05). Additionally, no statistically significant differences were found among the four models for 3-year LC prediction (*P>*0.05). A comparison of the AUC values for the 2-year and 3-year prediction models was illustrated in **Figures 2C, D**.

**Figure 3:**
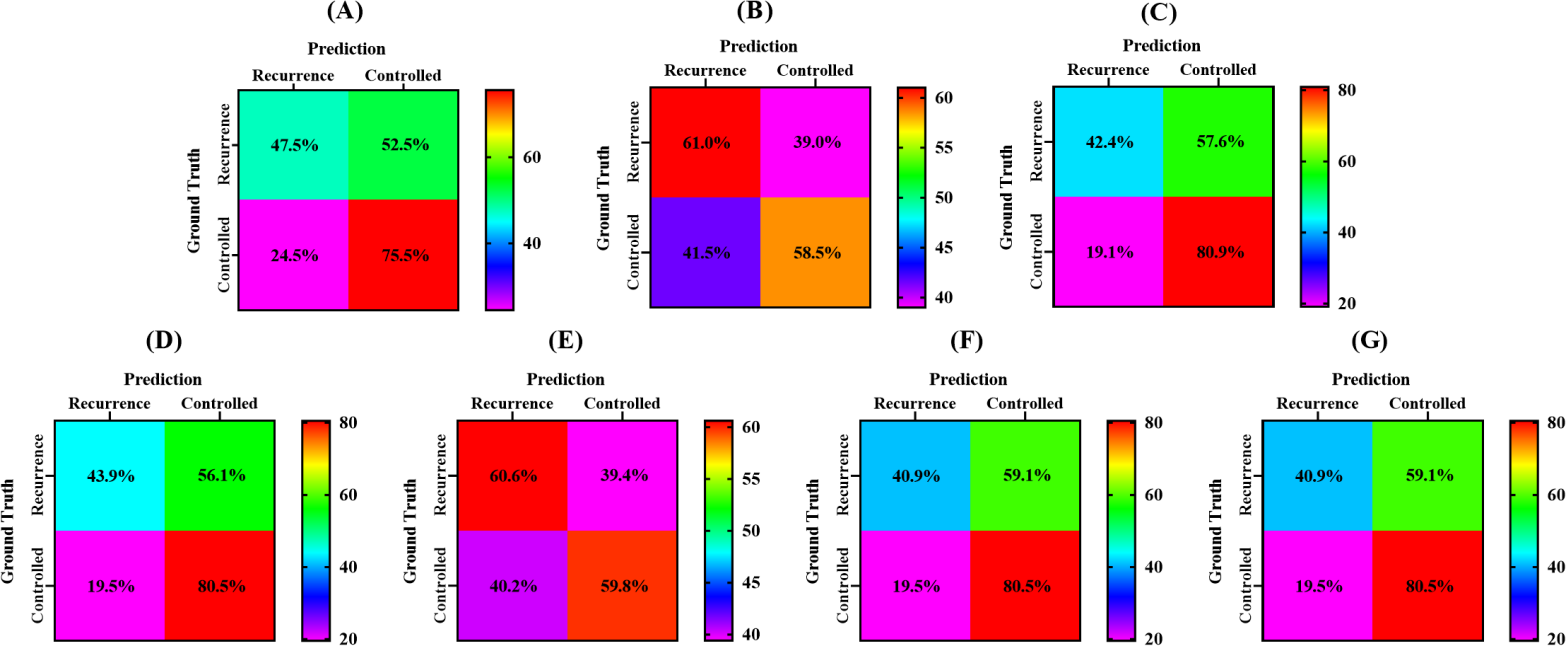
Confusion matrix of different radiobiological models. **(A)**, Liu model for 2-year LC prediction, **(B)**, Klement model for 2-year LC prediction, **(C)**, Ohri model for 2-year LC prediction, **(D)**, Liu model for 3-year LC prediction, **(E)**, Klement model for 3-year LC prediction, **(F)** Gucken model for 3-year LC prediction, and **(G)**, Santiago model for 3-year LC prediction.

The accuracy, sensitivity, and specificity values of the models were also tabulated in **Table 4**. **Figure 3** presented a confusion matrix that graphically represented the prediction accuracy within the radiobiological models.

**Figure 4:**
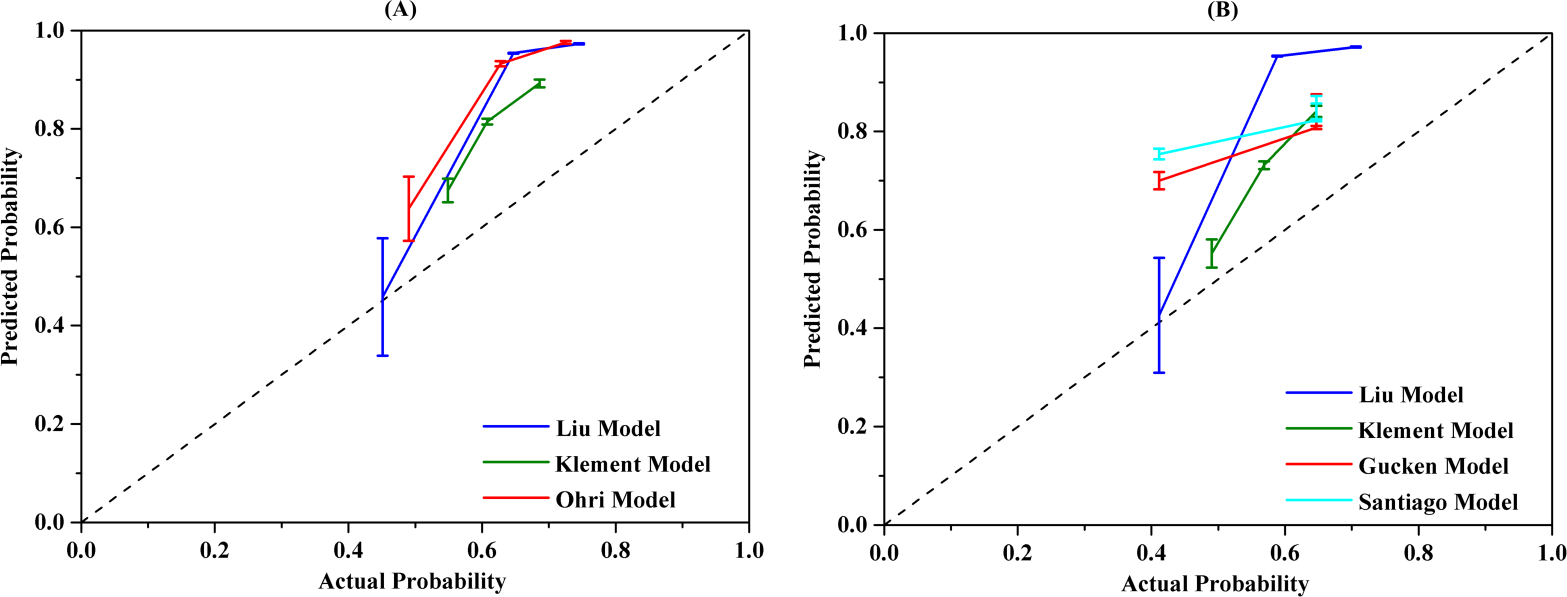
The calibration curves of different radiobiological models. **(A)** 2-year prediction model, **(B)** 3-year prediction model.

The calibration performance of the models was evaluated using a calibration plot to illustrate the accordance between the predicted probability and the observed values. The calibration plot is used to assess how well the predicted probabilities from a model correspond to the actual observed outcomes. If the model is perfectly calibrated, the calibration plot will lie along the 45-degree line, indicating that the predicted probabilities match well with the observed event rates. The accuracy of model calibration was carried out using the Hosmer-Lemeshow test and *P*>0.05 indicated no discrepancy between the observed and the predicted probabilities. The calibration curves for the models were displayed in **Figure 4**. The findings confirmed that the predicted probabilities from the models were consistent with the actual observations with *P*>0.05 when applying the Hosmer-Lemeshow goodness-of-fit test, with the exception of the Klement model for 2-year LC prediction, as noted in **Table 5**.

**Table 5:** Hosmer-Lemeshow goodness-of-fit test for the 2-year and 3-year prediction models.

Endpoint	Models	Chi-square	*p*-value
2-year LC	Liu Model	8.066	0.427
	Klement model	15.795	0.045
	Ohri model	3.890	0.867
3-year LC	Liu Model	7.685	0.465
	Klement model	12.877	0.116
	Gucken model	6.755	0.563
	Santiago model	6.810	0.557

LC, Local control.

### 3.5 Comprehensive comparison of different radiobiological models

A comprehensive analysis of different radiobiological models was shown in **Table 6**. The evaluation of the different models was based on several criteria, including their discriminative ability, calibration performance, clinical consistency, and the simplicity. Among the models for 2-year prediction, the Ohri model was identified as having strong discriminative power, good calibration performance, good agreement with clinical outcomes, and a relative simplicity when compared to the Liu model and the Klement model. For the 3-year models, both the Gucken model and the Santiago model demonstrated strong discriminative ability, good calibration performance, consistency with clinical outcomes, and a relatively simple methodology compared with the Liu model and the Klement model.

**Table 6:** A comprehensive comparison of the 2-year and 3-year models for LC prediction.

Endpoint	Models	Discrimination	Calibration	Clinical consistency	Model simplicity
2-year LC	Liu Model	Good	Good	Inconsistent	Two variables, Complicated calculation
	Klement model	Bad	Bad	Consistent	Six variables, Complicated calculation
	Ohri model	Good	Good	Consistent	Two variables, Simple calculation
3-year LC	Liu Model	Good	Good	Inconsistent	Two variables, Complicated calculation
	Klement model	Bad	Good	Consistent	Six variables Complicated calculation
	Gucken model	Good	Good	Consistent	Two variables, Simple calculation
	Santiago model	Good	Good	Consistent	Two variables, Simple calculation

LC, Local control.

Different models were compared according to their discriminative ability, calibration ability, clinical consistency and model simplicity.

## 4 Discussion

This study serves as the first study to externally validate the LC prediction of different radiobiological models in lung cancer patients treated with SBRT. Our results suggest that all the 2-year and 3-year models evaluated in the study showed potential discriminative ability, with the exception of the Klement model. Notably, the Ohri model demonstrated superior discriminative ability in comparison to the Klement model for predicting LC at 2 year. However, no statistically significant differences were observed among the four models when predicting 3-year LC outcomes. In the context of clinical practice, it is essential to consider both the accuracy and simplicity of a model when selecting a prediction tool. The Liu model is not recommended for use due to its complex mathematical computations and discrepancies with clinical observations. Likewise, the Klement model is also unsuitable because it incorporates multiple factors to predict the LC data, which compromises its simplicity. Taking into account all the factors discussed, we propose utilizing the Ohri model for 2-year LC prediction and either the Gucken model or the Santiago model for 3-year LC prediction. The models we endorse maintain a straightforward logistic relationship form while the prediction accuracy is not compromised.

Currently, the most traditional strategy for predicting LC is the application of BED_10_ > 100 Gy dose threshold (8). However, some studies have supported the recognition that tumor size also significantly impacts LC for NSCLC patients undergoing SBRT (19–21). Consequently, some radiobiological models that integrate dosimetric factors and tumor size have been proposed in recent years to enhance the prediction accuracy, with the 2-year LC or 3-year LC serving as the endpoints (8–13). The Ohri model, a logistic model, was developed by retrospectively analyzing 504 NSCLC tumors treated with various SBRT schedules. The model introduces the concept of size-adjusted biologically effective dose (sBED), which takes into account both tumor diameter and BED to predict the 2-year probability of LC following SBRT. Both the Gucken model and the Santiago model, which assume a sigmoidal dose-response relationship between TCP and BED, utilize only the isocenter dose as a predictor. Therefore, these three prediction models are fundamentally similar, with the primary distinction lying in the specific parameters used within each model. The Liu model, which considers the tumor regrowth after radiation treatment, can be employed to predict both 2-year and 3-year LC data based on the isocenter dose and tumor size. However, the accuracy of the model is a subject of further debate for two main reasons. Firstly, the model suggests drastically steep relationships between TCP and BED within the dose range of 50-60 Gy using a large α/β value of about 20 Gy, implying that TCP is nearly zero when the BED is below 50 Gy_20_. This may not correspond with clinical realities (13, 14, 22, 23). Secondly, it is a well-established fact that the LC rate for the tumor decreases significantly with extended follow-up periods (11, 24–26), however, it is puzzling that the Liu model predicts only a minor decrease in 3-year LC data compared to the 2-year LC data (**Table 3**). Additionally, the model involves complex mathematical calculation that might limit its clinical application. The Klement model, a Bayesian cure rate-based model, employs up to 6 factors to predict the LC probability, including isocenter dose, tumor size, gender, age, tumor location and whether chemotherapy was administered prior to SBRT. The identified limitation may compromise its clinical utility. Based on the aforementioned analysis and the results from external validation, we recommend to use the Ohri model for 2-year LC prediction and either the Gucken model or the Santiago model for 3-year LC prediction.

A deficiency in potential discriminative ability was observed with the Klement model, irrespective of its application for either 2-year or 3-year prediction. This result can be ascribed to two principal factors. Firstly, it is important to recognize that the radiobiological models used in the study are predominantly tailored for primary lung cancer, whereas the Klement model is specifically centered on pulmonary metastases. Nevertheless, we included the Klement model in our analysis because Guckenberger’s research emphasized that there is no significant difference in the dose-response relationship for local tumor control between primary lung cancer and lung metastases when treated with SBRT (27). Secondly, the Klement model incorporates up to six variables in its predictive approach. However, it is a widely accepted notion that including an excessive number of variables in a prediction model can result in poorer outcomes during external validation processes.

The findings of the study highlight the potential utility of radiobiological models in predicting the LC of lung cancer following SBRT, with statistically significant discriminatory ability and reliable calibration. However, it is crucial to observe that the highest AUC value among different models is only 0.625, which suggests a further need to improve the accuracy of these prediction models. One possible explanation for this may lie in the fact that radiobiological models predominantly concentrate on the effects of treatment dose and tumor size on LC. Recently, there has been a growing interest in the prognostic importance of inflammation-related factors, especially within the era of immunotherapy. Numerous independent studies have highlighted the correlation between these factors and overall survival (OS) in lung cancer patients who have undergone SBRT (28–31). However, the current literature regarding the prognostic significance of these factors in predicting LC following SBRT is quite limited. Further researches is indispensable to investigate the feasibility of integrating these factors into radiobiological models, thereby enhancing the accuracy of LC prediction for lung cancer patients.

The Ohri model illustrates a logistic correlation between TCP and both tumor size and treatment dose. Similarly, both the Gucken model and the Santiago model also exhibit a logistic relationship, specifically between TCP and the administered treatment dose. The clinical applicability of the three models is enhanced by their fewer number of variables, coupled with the accessibility and simplicity of their calculation methods. Conversely, the main obstacle for the adoption of the recommended models is their uncertainty on the net benefit, which necessitates a multicenter retrospective study to validate their efficacy. Even with potentially lower AUC values, we believe that the Ohri, Santiago, and Gucken models could be particularly suitable for two patient groups. The first group comprises elderly patients with comorbidities, who frequently face a multitude of health challenges. These models may facilitate more effective, personalized treatment strategies for this vulnerable demographic. The second group includes patients with large tumors, for whom predicting local recurrence is particularly challenging. These models comprehensively consider both the treatment dose and tumor size, thereby enhancing the precision of tumor control probability predictions. And this is notably true for the Ohri model, which highlights the effect of both tumor size and treatment dose on local control for the tumors.

In summary, the radiobiological models exhibited different prediction accuracy. Furthermore, some models did not align well with the clinical outcomes, and others employed complex mathematical computations. We recommend the use of the Ohri model for 2-year LC prediction and the Gucken model or the Santiago model for 3-year LC prediction. The models we recommend are simple at their logistic relationship form without compromising the predictive accuracy. Although we have validated the predictive accuracy of the radiobiological models using an SBRT database from external institution, the study has two limitations that must be acknowledged. Firstly, the is a single-institutional study and the sample size was relatively small, which could raise questions about the reliability and generalizability of external validation. Therefore, further multicenter retrospective investigation with a larger sample size is necessary for validation. Secondly, given the retrospective nature of the data collection, there may be inherent biases within the study that need to be considered. Thirdly, one might question whether including both primary and metastatic, as well as central and peripheral lung cancer patients, could potentially weaken our finding. Guckenberger et al. demonstrated that there was no difference in tumor local control with SBRT between primary lung cancer and lung metastases (27). Additionally, Park et al. found that tumor location did not correlate with worse overall survival, LC, or toxicity following SBRT for NSCLC (32). Based on the literature mentioned above, we believe that the inclusion of a mixture of primary and secondary, central and peripheral lung tumors is unlikely to have significantly impacted our results.

## 5 Conclusions

The study determined that all of the models designed for 2-year and 3-year prediction demonstrated potential discriminative ability, with the exception of the Klement model. In particular, the Ohri model showed an improved discriminative ability when compared to the Klement model for predicting LC at 2 year. Nonetheless, there were no statistically significant differences observed among the four models concerning the 3-year LC prediction. To maintain both the prediction accuracy and the simplicity of the model, it is recommended to use the Ohri model for predicting 2-year LC and either the Gucken model or Santiago model for 3-year LC in patients with lung cancer treated with SBRT.

## Data Availability

The original contributions presented in the study are included in the article/supplementary material. Further inquiries can be directed to the corresponding author.
